# Analysis of the mouse mutant *Cloth-ears* shows a role for the voltage-gated sodium channel *Scn8a* in peripheral neural hearing loss

**DOI:** 10.1111/j.1601-183X.2009.00514.x

**Published:** 2009-10

**Authors:** F E Mackenzie, A Parker, N J Parkinson, P L Oliver, D Brooker, P Underhill, V A Lukashkina, A N Lukashkin, C Holmes, S D M Brown

**Affiliations:** † MRC Mammalian Genetics UnitHarwell, OX11 0RD, UK; ‡ MRC Functional Genomics Unit, Department of Physiology, Anatomy and Genetics, Le Gros Clark Building, South Parks Road University of OxfordOxford OX1 3QX, UK; § Department of Statistics, Henry Wellcome Centre for Gene Function1 South Parks Road, Oxford, OX1 3 TG, UK; ¶ School of Life Sciences, University of SussexFalmer, Brighton, BN1 9QG, UK

**Keywords:** Deafness, mouse, paroxysmal motor, peripheral neural hearing loss, Scn8a, Nav1.6, tremor, VGSC

## Abstract

Deafness is the most common sensory disorder in humans and the aetiology of genetic deafness is complex. Mouse mutants have been crucial in identifying genes involved in hearing. However, many deafness genes remain unidentified. Using *N*-ethyl *N*−nitrosourea (ENU) mutagenesis to generate new mouse models of deafness, we identified a novel semi-dominant mouse mutant, *Cloth-ears* (*Clth*). *Cloth-ears* mice show reduced acoustic startle response and mild hearing loss from ∼30 days old. Auditory-evoked brainstem response (ABR) and distortion product otoacoustic emission (DPOAE) analyses indicate that the peripheral neural auditory pathway is impaired in *Cloth-ears* mice, but that cochlear function is normal. In addition, both *Clth/Clth* and *Clth/+* mice display paroxysmal tremor episodes with behavioural arrest. *Clth/Clth* mice also show a milder continuous tremor during movement and rest. Longitudinal phenotypic analysis showed that *Clth/+* and *Clth/Clth* mice also have complex defects in behaviour, growth, neurological and motor function. Positional cloning of *Cloth-ears* identified a point mutation in the neuronal voltage-gated sodium channel α-subunit gene, *Scn8a*, causing an aspartic acid to valine (D981V) change six amino acids downstream of the sixth transmembrane segment of the second domain (D2S6). Complementation testing with a known *Scn8a* mouse mutant confirmed that this mutation is responsible for the *Cloth-ears* phenotype. Our findings suggest a novel role for *Scn8a* in peripheral neural hearing loss and paroxysmal motor dysfunction.

Profound or severe hearing impairment affects around 1 in 1000 live births and another 1 in 1000 children suffer hearing loss before adulthood. Approximately 30% of humans over the age of 65 and 40–50% of humans over the age of 75 are affected by hearing loss [National Institute on Deafness and Other Communication Disorders (NIDCD, http://www.nidcd.nih.gov/)]. Defects in the external, middle and inner ears and the peripheral neural and central auditory processing systems can result in hearing impairment in mice and humans. Pathological causes include morphological defects of the middle and inner ears, developmental and functional defects of cochlear components and neural defects in peripheral audition and central auditory processing (Steel 1995). Genetic factors are estimated to cause around 50% of all deafness cases (Cohen & Gorlin 1995); therefore, uncovering the genetic pathways involved in hearing and deafness is crucial to the development of therapies that either prevent or correct deafness causing pathologies (Atar & Avraham 2005; Holley 2005). However, the aetiology of genetic deafness is complex. A large number of genes involved in hearing and/or deafness have been identified (see Hereditary Hearing Loss homepage: http://webh01.ua.ac.be/hhh/). Estimates vary but it seems likely that several hundred genes may be involved in the hearing process. Not only have mouse models have been crucial in identifying many of these genes, but in addition many mouse mutants have recapitulated the variety of pathologies, age of onset and mode of inheritance of human deafness (Brown *et al.* 2008). Comparison of human deafness loci and the map positions of known deaf mouse mutants shows that many loci do not have a comparative locus in the other species (Parkinson & Brown 2002), emphasizing that a large number of genes remain to be discovered. In particular, the genetics of peripheral neural hearing, central auditory processing, late-onset and noise-induced hearing loss is still poorly understood.

To identify novel genes involved in hearing loss, we used the resources of the UK *N*-ethyl *N*-nitrosourea (ENU) mouse mutagenesis programme (Nolan *et al.* 2000). One mutant generated from this screen, the *Cloth-ears* (*Clth*) mouse, was identified by its poor startle response to sound. We found that *Clth/Clth* mice have mild but discernable hearing loss [+12 dB sound pressure level (SPL) threshold shift]. Auditory-evoked brainstem response (ABR) and distortion product otoacoustic emission (DPOAE) analysis indicated that this hearing loss is of peripheral neural (retrocochlear) origin. Furthermore, the *Cloth-ears* mutant has additional motor symptoms. Mapping and candidate gene sequencing showed a novel mutation in the neuronal voltage-gated sodium channel (VGSC) α-subunit *Scn8a*, causing a non-synonymous change in the SCN8A peptide. The *Scn8a* gene is mutated in at least 11 mouse mutants, which show a range of motor dysfunction including paralysis, dystonia and tremor. However, mutation in *Scn8a* has never been shown to cause hearing impairment in either mice or humans. Here, we show that mutation in *Scn8a* is associated with peripheral neural hearing impairment and paroxysmal motor dysfunction in a novel mouse mutant and suggest a role for SCN8A in these processes.

## Methods

### Animals and complementation testing

Mice were kept on a 12-h-light, 12-h-dark cycle at 19–23°C and 45–65% humidity. Up to four mice were housed per cage. Mice were fed SDS 3ME (Special Diet Services, Witham, Essex, UK) pellets and chlorine sterilized water. All *Cloth-ears* mice were bred in-house and genotyped using standard polymerase chain reaction (PCR) methods once a genetic map position for *Cloth-ears* was established. The *Cloth-ears* founder arose on a BALB/cAnN × C3H/HeH genetic background. *Cloth-ears* mice were generated by backcross breeding of genotyped *Clth/+* to C3H/HeH or by intercross breeding of genotyped *Clth/+* mice. *In vitro* fertilization (IVF) procedures were carried out by the MRC Harwell Frozen Embryo and Sperm Archive (FESA) core. For complementation testing, cryopreserved sperm from C57BL/6J- *Scn8a*^4*J*^/J mice carrying the N1370T mutation in *Scn8a* was obtained from the Jackson Laboratory (stock number 004102) and rederived by IVF and maintained by backcross on the C57BL/6 strain. Several C57BL/6J- *Scn8a*^4*J*^/J/+ mice were mated to confirmed *Scn8a*^*Clth*^/+ mice, and the offspring from several litters were examined for abnormal phenotypes (including tremor, gait defects, hindlimb dragging and paralysis) every 2 days from birth until 21 days old, when mice were culled for welfare reasons.

### Genetic mapping

DNA extraction from mouse tissues and PCR amplification were performed under standard protocols. Annotated polymorphic markers were identified from public databases [microsatellite markers: Mouse Genome Informatics (MGI), Broad Institute Genetic Map of the Mouse Genome (Whitehead/MIT) and Center for Inherited Disease Research (CIDR); single nucleotide polymorphism (SNP) markers: Ensembl, Genomics Institute of the Novartis Research Foundation website (GNF)] and were tested for polymorphism in the parental strains BALB/cAnN and C3H/HeH. Polymerase chain reaction of microsatellite amplicons for low-resolution mapping was performed using one fluorescently labelled primer per reaction. Polymerase chain reaction products were analysed on 6% acrylamide gels on an ABI Prism 377 DNA sequencer and 377 XL DNA sequencer Data Collection (version 2.6) software. Results were analysed using ABI Prism GeneScan Analysis software (version 3.7.1) and ABI Prism Genotyper software (version 3.7). For high-resolution mapping, polymorphic microsatellite and SNP amplicons were analysed on 6% acrylamide gels using the single-stranded conformational polymorphism (SSCP) method and by pyrosequencing on a PSQ HS 96A pyrosequencer (Biotage) using one biotinylated primer per reaction. Primers used for the in-house marker *Scn8a_SNP* were forward: AGCTAATAAGCAAGGAGG; reverse: CACCTATGACTAAGCTAGCC and *Scn8a_SNP*: CAGCAGCC*N*CTATGTCTCTTT.

### Candidate gene assessment and mutation screening

Candidate genes mapping to the non-recombinant region were identified using the Ensembl mouse genome database (versions 30, 32 and 33) and assessed for candidacy by expression and function [MGI, GeneCards database, version 2.31 (Weizmann Institute of Science)], mouse mutants [MGI, Transgenic/Targeted Mutation database (TBASE)] and associated human disorders [Online Mendelian Inheritance of Man (OMIM)]. Exonic and splice-site sequence from candidate genes was amplified from genomic DNA from two *Clth/Clth* mice and the two parental strains BALB/cAnN and C3H/HeH, and was sequenced under standard protocols. Sequences were examined for integrity using the ABIPrism EditView ABI Automated DNA Sequence Viewer (version 1.0.1) viewer programme and analysed for mutation using DNAStar Lasergene (v6.0) sequence analysis software.

### Peptide predictions

Peptide sequences of mouse SCN8A, other mouse SCN-alpha peptides and SCN8A orthologues were obtained from Ensembl. Wild-type and mutant *Cloth-ears* peptide sequences were predicted and compared using EditSeq computer software (DNAStar). Other mouse SCN peptides and SCN8A orthologues were aligned using clustal-W web-based software (http://www.ebi.ac.uk/Tools/clustalw/). The position of the *Cloth-ears* mutant amino acid in the SCN8A protein was determined using putative predicted protein domains from Ensembl and UniProt databases.

### Analysis of auditory function

#### Assessment of the acoustic startle response

Clickbox (Institute of Hearing Research, Nottingham, UK) and quantitative startlebox (acoustic startle) (SmithKline Beecham Pharmaceuticals, plc.) protocols were carried out as outlined on the EMPRESS website (http://www.empress.har.mrc.ac.uk). During clickbox testing, a quick backwards flick of the ear pinna (the Preyer's reflex) and a rapid ‘jump’ or contraction of the neck and trunk muscles (the startle response) was recorded as a normal response. A lack of either the Preyer's reflex or startle response, or a markedly milder response of either reflexes, was recorded as a reduced response. A complete lack of both reflexes was recorded as no response. In startlebox (acoustic startle) analysis, the maximal startle amplitude and the latency of the maximal startle to 110 dB SPL, white noise, 40 ms tone bursts, and of background movement, was recorded. Mean gross startle amplitudes were calculated from 10 startle trials, mean basal movement was calculated from 10 recordings in the absence of a soundburst and mean startle amplitudes were calculated by subtracting the mean basal movement from the mean gross startle amplitude for each mouse. Amplitude and latency of the startle response of mice was measured using Windows 3.1 software.

#### Auditory-evoked brainstem response (ABR) analysis

Mice were anaesthetized with ketamine (Ketaset™) and additionally given medetomidine (Domitor™) for muscle relaxation and analgesia by intraperitoneal injection (0.5 ml Domitor™ at 100 mg/ml with 4.12 ml water and 0.38 ml Ketaset™ at 1 mg/ml; administered at a rate of 0.1 ml/10 g of body weight). Animals were placed in an audiometric chamber (IAC 401-A-SE) on a heated mat (∼ 37°C) to maintain body temperature. Acoustic stimuli were delivered monaurally to the right ear at a distance of 1.5 cm via a free field transducer [ES1 Tucker Davis Technology (TDT), Alachua, FL, USA], controlled by SigGen/BioSig software (TDT), using TDT system III hardware. The transducer was calibrated using a 1 4” measuring microphone (7016 ACO-Pacific, Belmont, CA, USA) and SigCal software (TDT). Electrodes (Grass Telefactor F-E2-12) were placed subdermally over the vertex (active), right mastoid (reference) and left mastoid (ground). The response from the electrodes was recorded for a period of 10 ms, over a total of 312 repetitions, and amplified using TDT system III hardware. The repetitions were averaged and bandpass filtered between 300 and 1500 Hz, using the BioSig software. Tone burst stimuli totalled 7 ms duration including 1 ms rise/fall time, and were presented at a rate of 21/s starting at 90 dB SPL followed by decreasing steps of 10 dB until 50 dB SPL, and in 5 dB steps from 45 to 5 dB SPL, until a threshold was reached. Threshold estimation was determined visually by the lack of replicable peak morphology in the response trace. Frequencies used were tone bursts at 8, 12, 20 and 26 kHz for threshold determination and at 8, 12, 20 and 32 kHz for analysis of peak latency. Recovery of mice was accelerated by administration of atipamezole (Antisedan™, 5 mg/ml) at the rate of 1 mg/kg of body weight.

#### Distortion product otoacoustic emission (DPOAE) analysis

Mice were anesthetized with ketamine (0.12 mg per g of body weight, i.p.) and xylazine (0.01 mg per g, i.p.). Sound was delivered via a probe with its tip within 1 mm of the tympanic membrane and coupled to a closed acoustic system comprising two MicroTech Gefell 1 inch MK102 microphones for delivering tones and a Bruel & Kjaer 3135 1/4 inch microphone for monitoring sound pressure at the tympanum. The sound system was calibrated *in situ* in dB SPL for frequencies between 1 and 70 kHz. The position of the coupler was adjusted to minimize peaks and dips in the calibration curve. White noise and tone pulses with rise-fall times of 0.2 ms were synthesized by a Data Translation 3010 data acquisition board, attenuated and used for sound system calibration and acoustic stimulation. Data were digitized at 250 kHz and stored on a PC. Distortion product otoacoustic emissions were measured for levels of *f*_1_ ranging from 10 to 80 dB SPL, with the levels of the *f*_2_ tone set 10 dB below that of the *f*_1_ tone. Distortion product otoacoustic emission threshold curves were constructed from measurements of the level of the *f*_1_ tone that produced a 2*f*_1_–*f*_2_ DPOAE with a level of 0 dB SPL, where the frequency ratio of *f*_2_/*f*_1_ was 1.23. System distortion during DPOAE measurements was 80 dB below the primary tone levels.

### Histological analysis

All mice examined by histological methods were sex matched with littermate controls. Ossicles were dissected, placed into formalin solution and examined by X-ray analysis using Faxitron equipment (Qados Ltd, Sandhurst, Berkshire, UK). For examination of cochlear hair cells, brains were removed and bisected heads were placed into 2.5% glutaraldehyde in 0.1 m phosphate buffer. Cochleae were dissected and fixed in fresh 2.5% glutaraldehyde in 0.1 m phosphate buffer for 4–5 h, rotating, at 4°C, and then washed four times for 5 min in 0.1 m phosphate buffer. The bony shell of the cochlea was then removed. Samples were processed by dehydrating, critical point drying and gold sputter coating at the Electron Microscopy Unit, Royal Holloway University of London, Egham, Surrey TW20 0EX. Ultrastructural analysis of cochlear hair cells was carried out using Hitachi S-2400 and S-3000N scanning electron microscopes. Necropsy and histological analysis was carried out as outlined on the EMPReSS website (http://www.empress.har.mrc.ac.uk). For cerebellar histopathology, brains were removed and snap-frozen on dry ice in Optimal Cutting Temperature (OCT) mounting medium (VWR). Fifteen micrometre parasagittal cryosections were cut on a microtome, mounted on Superfrost Plus slides (BDH) and stained in 0.5% cresyl violet solution. For cell counts and measurements, 5 μm coronal wax sections from fixed brains were taken every 100 μm through the cerebellum and stained with cresyl violet. Five matched sections (spanning 400 μm) were used for Purkinje cell counts and measurements at three regions in the anterior (containing predominantly lobes I–VI), posterior (lobes VII–IX) and midway between these selected regions, posterior to the primary fissure (lobes VI and X). Cells were counted if the nuclei could be visualized. Maximum width of the sections (discounting the paraflocculus) and distance from the top of the cerebellum to the fourth ventricle or medulla at the base of the cerebellar vermis were also measured. The ratio between the width Purkinje cell layer (PCL) and granule cell layer (GCL) was calculated by measuring matched regions in the cerebellar hemispheres in all sections.

### Phenotypic and behavioural testing

**Figure 5:**
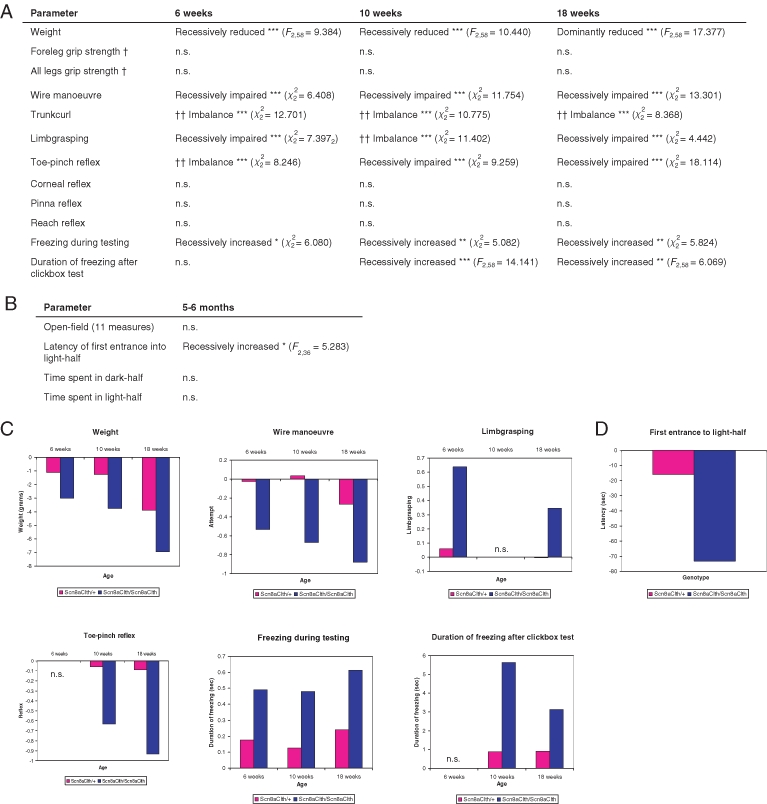
(a) Statistical analysis for genotypic differences of weight, neurological, neuromuscular and behavioural phenotypes of *Scn8a*^*Clth*^ mice at 6, 10 and 18 weeks (*n* = 73), where *F*_t,u_ denotes the *F*-statistic on t degrees of freedom. Continuous numeric data were analysed under linear models, binary data using generalized linear models and ordinal data using proportion odds logistic regression. Phenotypes significantly different to wild types are given with the best fit of inheritance. n.s., not significant; *P < 0.05; **P < 0.01; ***P < 0.001 (unadjusted *P* values). † = Only +/+ and *Scn8a*^*Clth*^*/Scn8a*^*Clth*^ were tested. †† = Where an imbalance of design confounded statistical analysis, manual analysis of individual data points showed that *Scn8a*^*Clth*^*/Scn8a*^*Clth*^ displayed increased trunk curling and limb grasping and impaired toe-pinch reflex compared with other genotypes. (b) Statistical analysis for genotypic differences of anxiety and locomotion behaviour of *Scn8a*^*Clth*^ mice at 5–6 months old (*n* = 39), where *F*_t,u_ denotes the *F*-statistic on t degrees of freedom. Tests were performed using the open-field and light-dark box behavioural paradigms. Analysis was performed under a linear model. Phenotypes significantly different to wild types are given with the best fit of inheritance. n.s., not significant; *P < 0.05; **P < 0.01; ***P < 0.001 (unadjusted *P* values). (c) Graphical representations of significantly different weight, neurological, motor and behavioural phenotypes in *Scn8a*^*Clth*^ mice, showing data for adjusted *P* values < 0.05. Data shown are estimate values for *Scn8a*^*Clth*^/+ and *Scn8a*^*Clth*^/*Scn8a*^*Clth*^ mice, having adjusted for sex and mating identifier, where +/+ is normalized to = 0. n.s., not significant. (d) Graphical representation of significantly different anxiety/locomotor-related phenotypes in *Scn8a*^*Clth*^ mice compared with wild types, showing data for adjusted *P* values < 0.05.. Data shown are estimate values for *Scn8a*^*Clth*^/+ and *Scn8a*^*Clth*^/*Scn8a*^*Clth*^ mice, having adjusted for sex and mating identifier, where +/+ is normalized to = 0. n.s., not significant

A cohort of 73 +/+, *Scn8a*^*Clth*^/+ and *Scn8a*^*Clth*^/*Scn8a*^*Clth*^ mice was generated by mating nine *Scn8a*^*Clth*^/+ siblings [generation (N5xN5)N2] in six trio matings and comprised of all mice born over several months. Phenotypic analysis was carried out using a modified SHIRPA protocol, adapted from Rogers *et al.* (1997). (a similar protocol can be found at http://www.empress.har.mrc.ac.uk). Additional phenotyping analysis included (1) specific observations of mouse general behaviour over 1 min (assessing the presence of facial twitches, muscle spasms of the body, piloerection, shaky or unsteady movement, abnormal postures, repetitive nosepoking, hyperactivity, excess rearing); (2) fore- and back-leg grip strength using a grip strength meter (BioSeb) and (3) excess freezing behaviour occurring outside the viewing jar and the duration of freezing after the clickbox test. Due to welfare issues concerning *Scn8a*^*Clth*^/*Scn8a*^*Clth*^ mice, only +/+ and *Scn8a*^*Clth*^/+ mice were tested in the grip strength test (*n* = 56). Scoring of tests shown in Fig. 5 was performed as follows: wire manoeuvre: good attempt = 1, impaired attempt = 0; limbgrasping: absent = 0, present = 1; toe-pinch reflex: absent = 0, present = 1 and freezing during testing: absent = 0, present = 1. Thirty nine +/+, *Scn8a*^*Clth*^/+ and *Scn8a*^*Clth*^/*Scn8a*^*Clth*^ littermate mice from the cohort were tested in the open-field and light-dark behavioural tests (five females and eight males per genotype). Open-field testing was performed as outlined on the EMPReSS website (http://www.empress.har.mrc.ac.uk) using videotracking software analysis by EthoVision; mice were scored for latency of first movement in arena (seconds), number of movements into the centre, duration spent in centre (seconds), distance moved in arena (cm) and in centre (cm), maximum distance per move in arena (cm) and in centre (cm), mean velocity in arena (cm/seconds) and in centre (cm/seconds), duration of moving in arena (seconds) and in centre (seconds). Light-dark apparatus and software used was TruScan 99 (Coulbourne Instruments). Tests were performed between 0900 and 1400 h. Mice were tested in the same order as the open-field test. Mice were moved from the home room into a quiet acclimatization room at least 30 min before the start of the test. A clean, opaque Perspex dark box was placed into the left-hand side of the clean arena with the opening facing to the left. Red light only was used for illumination using a lamp directly above the ‘lit’ region of the arena. Mice were placed directly from the home cage into the opening of the dark box, and the recording was started. The time for the mouse to enter the light half of the arena (scored by all four paws placed in the light half) was recorded manually and the mouse was left undisturbed to complete the test.

### Statistical analysis

Except for ABR threshold data, which were analysed under a two-tailed Student's *t*-test, statistical analysis was carried out using the software package R (2005). Data was statistically analysed for a genotype effect. To adjust for confounding variables, continuous numeric data were analysed under a linear model using analysis of variance (anova) and multivariate *F*-test; binary data were analysed under a generalized linear model and ordinal data were analysed under a proportional odds logistic regression model. The significance of a genotype effect was tested under a model which accounted for other factors, namely, (mating identifier, sex, genotype) vs. (mating identifier, sex). Data obtained by using different mouse cohorts were analysed separately. Tests showing an ‘imbalance of design’ [where there was a large discrepancy in the number of individuals showing different responses within a test, e.g. in a binary (qualitative) test where 65 mice showed a positive response and 8 mice showed a negative response] were manually analysed for the distribution of values by genotype. *P* values were corrected for multiple hypotheses testing using the false discovery rate test (FDR, 5%). Estimate values for *Scn8a*^*Clth*^/+ and *Scn8a*^*Clth*^/*Scn8a*^*Clth*^ mice (relative to +/+) under this model were obtained, having accounted for sex and mating identifier. Tests showing significance (FDR = 5%) were further tested to find the best fit of mode of inheritance, under a ‘dominant’ model [(mating, sex, non-wild-type status, homozygous status) against (mating, sex, non-wild-type status)] and a ‘recessive’ model [(mating, sex, non-homozygous status, wild-type status) against (mating, sex, non-homozygous status)].

## Results

### Identification of the Cloth-ears mouse mutant

The *Cloth-ears* mouse mutant was identified in a dominant mouse ENU-mutagenesis screen (Nolan *et al.* 2000). The founder mutant presented at 5 weeks with a reduced startle response to a 90 dB SPL, 20 kHz single tone burst, generated by a clickbox. Frozen sperm from the founder mouse was used to fertilize C3H/HeH oocytes by IVF. Startle response in offspring was assessed by clickbox test. We found that 30% of N3 offspring (*n* = 66) showed a mild or absent Preyer's reflex and a mild or absent startle response, confirming the genetic basis of this phenotype. A colony of *Cloth-ears* mice was generated for genetic mapping and phenotypic analysis by backcross of confirmed *Clth/+* mice to the C3H strain. Phenotype analysis of an N4 generation backcross cohort showed that the reduced startle response was autosomally inherited, but at reduced penetrance (16 of 58 backcross mice). In addition, *Clth/+* mice showed an episodic tremor (see below). *Cloth-ears/+* mice with a reduced startle response were intercrossed to produce *Clth/Clth* mice. Putative *Clth/Clth* mice displayed a continuous tremor (see below) and also displayed a reduced startle response to the clickbox, but at much greater penetrance (5 of 23 intercross mice). *Clth/+* and *Clth/Clth* mice showed a normal lifespan, no unexpected lethality and were fertile. The severity of continuous tremor in *Clth/Clth* mice and the frequency and severity of episodic tremor in *Clth/+* mice appeared to increase moderately with age (< 2 years), but neither *Clth/*+ or *Clth/Clth* mice developed gait defects, limb weakness or paralysis at any age.

### Genetic mapping and identification of the Cloth-ears gene

**Figure 1:**
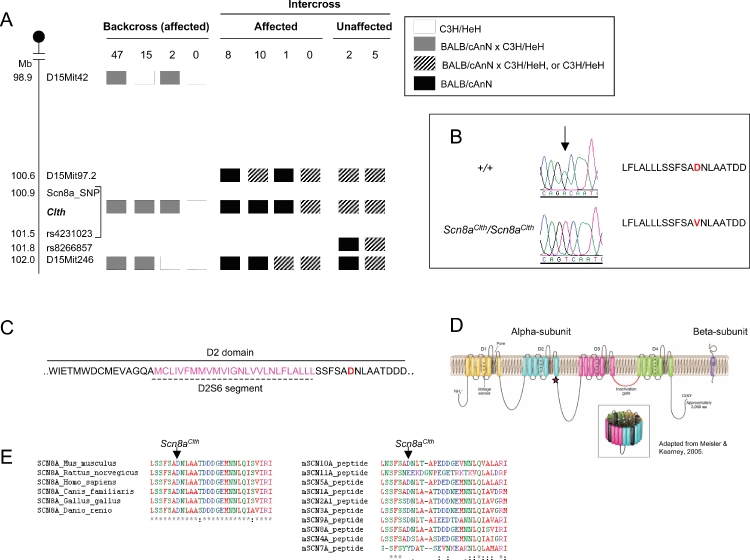
(a) Haplotypes of backcross and intercross progeny segregating the *Cloth-ears* mutation. The *Cloth-ears* (*Clth*) mutation arose on a mutagenized (BALB/cAnN × C3H/HeH)F1 individual. Sixty-four backcross progeny with a reduced startle response and/or an intermittent tremor and 26 intercross progeny with or without a continuous tremor were mapped across chromosome 8 and genotyped at recombinant markers. The genetic data indicates that the mutagenized chromosome was from the BALB/cAnN strain. The *Cloth-ears* mutation is non-recombinant with the SNP markers *Scn8a_SNP* and *rs4231023* and maps to the interval *D15Mit97.2–rs8266857*. (b) Chromatograms of the *Cloth-ears* mutation from wild-type (+/+) and *Scn8a*^*Clth*^/*Scn8a*^*Clth*^ DNA. Arrow: base 2942 of the *Scn8a* coding sequence, showing an adenine (A) in wild-type mice and a thymine (T) in *Scn8a*^*Clth*^/*Scn8a*^*Clth*^ mice (arrow). The predicted amino acid sequence from wild-type and *Scn8a*^*Clth*^/*Scn8a*^*Clth*^ mice is also indicated, highlighting the D981V change. (c) Position of the *Cloth-ears* mutation (D981V) in the SCN8A amino acid sequence. D981V resides within the predicted domain 2 (D2) of the SCN8A peptide, six amino acids downstream of the S6 transmembrane segment. The amino acid sequence of domain 2 (D2) is shown in black; sequence of domain 2 segment 6 (D2S6) is shown in pink and the position of the *Cloth-ears* mutation (D981V) is shown in bold red. (d) Schematic of the voltage-gated sodium channel α- and β-subunit proteins (adapted from Meisler & Kearney 2005). The α-subunit comprises four homologous domains (D1–D4 or DI–DIV), which are shown in different colours, each containing six transmembrane segments (S1–S6). S4 segments function as voltage sensors and the S5–S6 loops form the outer pore. A pore structure is formed by the transmembrane segments in the membrane (inset). The location of the *Cloth-ears* mutation is shown (red star). (e) Alignment of peptide sequences of SCN8A orthologues and other mouse SCN α-subunits (Ensembl) in the region of amino acid 981 (arrow). Alignments were made using clustal-W web-based software.

The *Cloth-ears* mutation arose on a mutagenized (BALB/cAnN × C3H/HeH)F1 individual. Marker analysis of 64 affected *Clth/+* backcross progeny allowed us to localize the *Cloth-ears* mutation to a 3.1 Mb region on distal chromosome 15 between the microsatellite markers *D15Mit42* and *D15Mit246* (Ensembl, version 50) (Fig. 1a), and indicated that the mutagenized chromosome was from the BALB/c strain. Moreover, genetic analysis of *Clth/Clth* mice derived from *Clth/+* intercrosses and showing a continuous tremor refined the non-recombinant region to 1.37 Mb between the markers *D15Mit97.2* and *D15Mit246*. Additional genotyping of non-affected (non-tremoring) mice from *Clth/+*×*Clth/+* intercrosses yielded two more recombinants that further refined the region to 1.2 Mb between the markers *D15Mit97.2* at 100.63 Mb and *rs8266857* at 101.83 Mb (Fig. 1a). The *Cloth-ears* locus was non-recombinant with the marker *rs4231023* and the in-house marker *Scn8a_SNP*.

The *Cloth-ears* non-recombinant region contains 33 genes, including a cluster of keratin genes and several novel genes. We selected candidate genes on the basis of their reported function, expression or association with known mouse mutants or human disorders. Two genes were initially selected as good candidates. The type 1 keratin 8 gene (*Krt2-8*, *Krt8*) is a component of the cytoskeleton localized to the supporting cells of the cochlear organ of Corti in adult mice (Kuijpers *et al.* 1992; Mogensen *et al.* 1998). The VGSC α-subunit type VIII gene (*Na*_*v*_1.6, *Scn8a*) is expressed in cerebellum, cortex and the peripheral nervous system (Burgess *et al.* 1995) and several *Scn8a* mouse mutants display intention tremor and gait defects (e.g. Kohrman *et al.* 1996; Sprunger *et al.* 1999). However, no hearing loss has been reported for *Scn8a* mouse mutants.

Mutation screening of coding sequence, intron–exon boundaries and 3’ untranslated regions of the candidate genes was carried out by sequencing genomic DNA from two *Clth/Clth* mice. Sequences were compared to genomic DNA sequence from both parental strains (BALB/cAnN and C3H/HeH). Ninety-five percent of *Scn8a* and 90% of *Krt2-8* coding sequences were analysed for mutations. An adenine to thymine tranversion was identified at base 2942 (A2942T) of the *Scn8a* coding sequence in *Clth/Clth* mice, which was not present in the parental strains (Fig. 1b). Genotyping of a cohort of *Clth/+* and *Clth/Clth* mice showed that this mutation was 100% concordant with *Cloth-ears* phenotype (*n* = 122). This base change causes a non-synonymous amino acid change from aspartic acid to valine at residue 981 of the SCN8A peptide (D981V) (Fig. 1b,c). Residue D981 is conserved in orthologous SCN8A peptides (Ensembl) (Fig. 1e). Residue D981 is also conserved in mouse SCN1A, SCN2A1, SCN3A, SCN4A, SCN5A, SCN9A and SCN10A, but not in SCN7A and SCN11A (Ensembl) (Fig. 1e). Residue D981 lies six amino acids downstream of the predicted end of the sixth transmembrane segment of the second domain in SCN8A peptide (D2S6) (Fig. 1d). This high level of conservation and position very near to a transmembrane domain suggests an important role for D981 in sodium channel function.

### Cloth-ears does not complement a known mutant of Scn8a

To assess whether the D981V mutation in SCN8A was causing the *Cloth-ears* phenotype, we carried out a complementation test cross with *Scn8a*^4*J*^, a known mouse mutant of *Scn8a. The Scn8a*^4*J*^ mutation is a missense mutation (Buchner *et al.* 2004). However, *Scn8a*^4*J*^*/Scn8a*^4*J*^ mice display a much more severe phenotype, showing hindlimb paralysis and juvenile death. *Scn8a*^4*J*^/+ mice are not reported to show any abnormal phenotype (Buchner *et al.* 2004). *Clth/+* mice were crossed to *Scn8a*^4*J*^/+ mice. Of 15 offspring, 3 showed a continuous tremor in the head and body (20%), beginning at 8–9 days old. Extended freezing and severe tremor episodes were noticeably present in one mouse. Interestingly, these mice also showed an abnormal gait. This result shows that *Cloth-ears* and *Scn8a*^4*J*^ do not complement: *Clth/+*, *Scn8a*^4*J*^/+ mice show a phenotype that clearly resembles the *Clth/Clth* phenotype, but that is slightly more severe. The *Cloth-ears* mutant will be therefore referred to as an allele of *Scn8a*, *Scn8a*^*Clth*^.

### Cloth-ears mice have a reduced acoustic startle response

**Figure 2:**
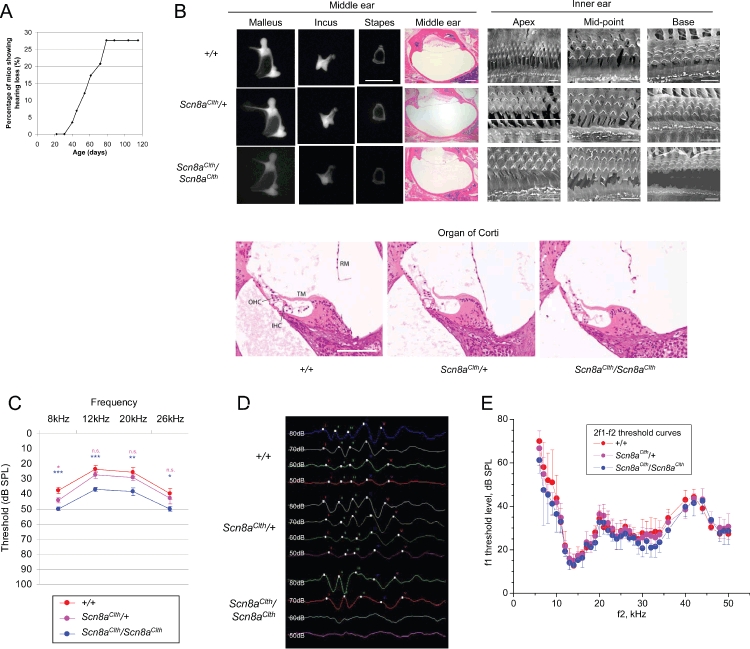
(a) Age of onset and penetrance of reduced startle response in *Scn8a*^*Clth*^ mice. Startle response analysis of 58 age-matched, N4 mice from a (*Clth/+*× +/+) cross, comprising ungenotyped *Scn8a*^*Clth*^/+ and +/+ progeny, showed that 27.6% of the cohort display a reduced startle response to a 90 dB SPL, 20 kHz single tone burst, indicating reduced penetrance. As mice are ungenotyped, these data include both +/+ and *Clth/+* mice. The onset of reduced startle response was between 31 and 79 days. (b) Representative histology of middle and inner ears of *Scn8a*^*Clth*^ mice. No morphological differences in structure, size or bone density of middle ear ossicles from 6–10-month-old *Scn8a*^*Clth*^/+ and *Scn8a*^*Clth*^/*Scn8a*^*Clth*^ mice were seen (scale bar, 1 mm). The small protusion on the stapes seen in the +/+ sample has detached with the incus during dissection in the *Scn8a*^*Clth*^/+ sample. H&E-stained 8 μm dorsal sections of the middle ear of 8-month-old *Scn8a*^*Clth*^/+ and *Scn8a*^*Clth*^/*Scn8a*^*Clth*^ mice shows that the middle ear cavity is free from effusive matter and the epithelium is smooth, indicating the absence of otitis media, and the tympanic membrane is intact (scale bar, 500 μm). H&E-stained 5 μm cochlear sections from 6-month-old *Scn8a*^*Clth*^/+ and *Scn8a*^*Clth*^/*Scn8a*^*Clth*^ mice shows a normal organ of Corti (OHC, outer hair cell; IHC, inner hair cell; TM, tectorial membrane; RM, Reissner's membrane; scale bar, 100 μm). In some samples, Reissner's membrane has detached during processing. Scanning electron microscopy of the exposed organ of Corti from 6- to 7-month-old *Scn8a*^*Clth*^/+ and *Scn8a*^*Clth*^/*Scn8a*^*Clth*^ mice shows three rows of OHCs and IHCs in all samples (scale bar, 8 μm). No patterning abnormalities or degeneration of stereocilia or hair cells was seen. Some artefacts of sample preparation can be seen (spherical blebs) in +/+ and mutant samples. (c) Mean and SEM of ABR hearing thresholds of 3-month-old sex-matched +/+ (*n* = 7), *Scn8a*^*Clth*^/+ (*n* = 7) and *Scn8a*^*Clth*^/*Scn8a*^*Clth*^ (*n* = 8) mice at 8, 12, 20 and 26 kHz. Thresholds of *Scn8a*^*Clth*^/+ mice were significantly different from +/+ mice at 8 kHz (*Scn8a*^*Clth*^/+ vs. +/+: 8 kHz, *P* = 0.0363; 12 kHz, *P* = 0.2570; 20 kHz, *P* = 0.3288 and 26 kHz, *P* = 0.5602). Thresholds of *Scn8a*^*Clth*^*/Scn8a*^*Clth*^ mice were significantly different from +/+ mice across all four frequencies tested (*Scn8a*^*Clth*^*/Scn8a*^*Clth*^ vs. +/+: 8 kHz, *P* = 0.0007; 12 kHz, *P* = 0.0006; 20 kHz, *P* = 0.0071 and 26 kHz, *P* = 0.0342). *Scn8a*^*Clth*^*/Scn8a*^*Clth*^ mice showed an average threshold increase of 10–14 dB SPL. Significance levels are indicated in pink for *Scn8a*^*Clth*^/+ and in blue for *Scn8a*^*Clth*^*/Scn8a*^*Clth*^. n.s., not significant; *P < 0.05; **P < 0.01; ***P < 0.001. (d) Representative ABR traces from 5–7-month-old sex-matched +/+ (*n* = 5), *Scn8a*^*Clth*^/+ (*n* = 5) and *Scn8a*^*Clth*^/*Scn8a*^*Clth*^ (*n* = 5) mice at 80, 70, 60 and 50 dB SPL to 8 kHz tones. Peaks 1–5 are labelled I–V. ABR of *Scn8a*^*Clth*^/*Scn8a*^*Clth*^ mice shows a merged morphology of peaks 3 and 4, and peak 4 shows a smaller amplitude than peak 3. Peaks 3–4 and 4–5 interpeak latencies appear prolonged. At 60 and 50 dB SPL, a response is seen from *Scn8a*^*Clth*^*/Scn8a*^*Clth*^ mice, but peaks are much less defined than from +/+ and *Scn8a*^*Clth*^/+ mice. (e) Threshold curves (mean ± SD ) for DPOAE at frequency 2*f*_1_–*f*_2_. The horizontal axis is *f*_2_ frequency. The vertical axis is the level of *f*_1_ to generate emission of 0 dB SPL. Level of *f*_2_ was 10 dB below the *f*_1_ level. Ratio *f*_2_/*f*_1_ was 1.23. Red symbols represent mean values for +/+ (*n* = 3), magenta symbols represent mean values for *Scn8a*^*Clth*^/+ (*n* = 6) and blue symbols represent mean values for *Scn8a*^*Clth*^*/Scn8a*^*Clth*^ (*n* = 4) mice. There was no significant difference between the three genotypes at any frequency tested

**Figure 3:**
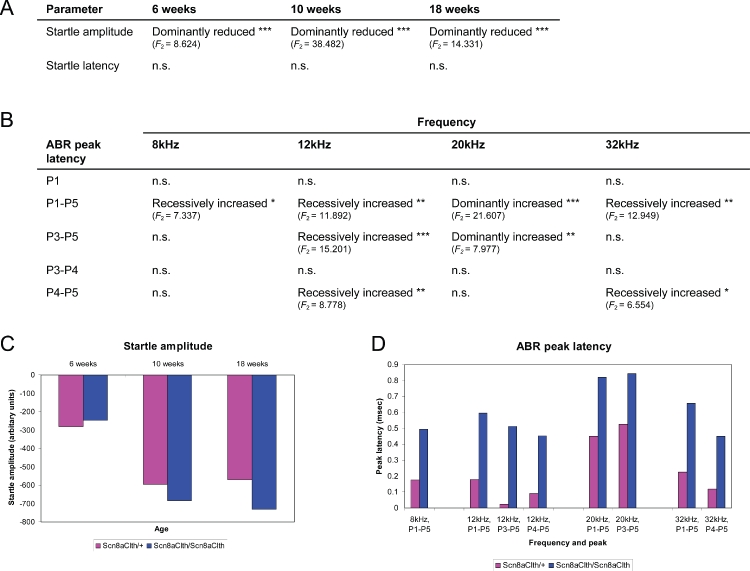
(a) Statistical analysis for genotypic differences of hearing phenotypes of *Scn8a*^*Clth*^ mice at 6, 10 and 18 weeks (*n* = 73), where *F*_t,u_ denotes the *F*-statistic on t degrees of freedom. Analysis was performed under a linear model (see *Methods*). Startle amplitudes and maximal latencies were measured in response to 40 ms soundbursts of white noise at 110 dB SPL using the startlebox paradigm. Parameters significantly different to wild types are given with the best fit of inheritance. n.s., not significant; *P < 0.05; **P < 0.01; ***P < 0.001 (unadjusted *P* values). (b) Statistical analysis for genotypic differences of ABR peak latencies of *Scn8a*^*Clth*^ mice at 5–7 months old, where *F*_t,u_ denotes the *F*-statistic on t degrees of freedom. Analysis was performed under a linear model (see *Methods*). Parameters significantly different to wild types are given with the best fit of inheritance. n.s., not significant; *P < 0.05; **P < 0.01; ***P < 0.001 (unadjusted *P* values). (c and d) Graphical representation of significantly different startle response and ABR peak latency parameters in *Scn8a*^*Clth*^ mice compared with wild types, showing data for adjusted *P* values < 0.05. Startle response was measured by placing mice in a soundproof chamber containing a loudspeaker to generate sound. Startle amplitude was calculated in arbitary units using an accelerometer connected to the chamber floor to measure floor displacement upon sound presentation. Data shown are estimate values for *Scn8a*^*Clth*^/+ and *Scn8a*^*Clth*^/*Scn8a*^*Clth*^ mice, having adjusted for sex and mating identifier, where +/+ is normalized to = 0.

We assessed in more detail the onset of startle response impairment in *Scn8a*^*Clth*^/+ mice by weekly clickbox tests of mice from an age-matched backcross cohort (*n* = 58). The onset of a persistently reduced startle response was found to be between 31 and 79 days (Fig. 2a). We assessed the impairment of the startle response quantitatively using startlebox analysis. At 6, 10 and 18 weeks old, both *Scn8a*^*Clth*^/+ and *Scn8a*^*Clth*^*/Scn8a*^*Clth*^ mice showed significantly reduced amplitude of startle response to white noise at 110 dB SPL. Statistical analysis showed that the reduction in startle best fit a dominant model (see *Methods*; Fig. 3a,c). This result confirmed the reduced startle response that was observed in *Scn8a*^*Clth*^ mice by clickbox test. In addition, the startle amplitude progressively worsened between 6 and 10 weeks old (Fig. 3c). This suggests that the impairment of startle in *Scn8a*^*Clth*^ mice begins before 6 weeks of age and that this impairment progresses until at least 10 weeks of age, supporting the previous findings from weekly clickbox tests. No difference in latency of peak startle amplitude was found at 6, 10 or 18 weeks of age in either *Scn8a*^*Clth*^/+ or *Scn8a*^*Clth*^*/Scn8a*^*Clth*^ mice (Fig. 3a). A residual startle response in almost all mice tested indicated that neither *Scn8a*^*Clth*^/+ or *Scn8a*^*Clth*^*/Scn8a*^*Clth*^ mice were profoundly deaf. However, this analysis did not rule out the possibility of a motor impairment, instead of hearing loss, as the primary cause of the reduced startle response. In addition, *Scn8a*^*Clth*^/+ or *Scn8a*^*Clth*^*/Scn8a*^*Clth*^ mice did not show evidence of vestibular impairments such as circling, headbobbing or abnormal behaviour in grid walking or swimming (data not shown).

### Cloth-ears mice display ABR abnormalities indicative of a peripheral neural auditory defect

We examined *Cloth-ears* mice for hearing loss by ABR analysis. To determine auditory thresholds, ABR analysis was performed on sex-matched 3-month-old +/+, *Scn8a*^*Clth*^/+ and *Scn8a*^*Clth*^*/Scn8a*^*Clth*^ mice (*n* = 22) at decreasing dB SPL levels from 90 dB SPL at 8, 12, 20 and 26 kHz (Fig. 2c). Statistical analysis showed that auditory thresholds of *Scn8a*^*Clth*^/+ mice were only significantly increased compared with +/+ mice at 8 kHz, but that *Scn8a*^*Clth*^*/Scn8a*^*Clth*^ thresholds were significantly increased compared with +/+ mice across the four frequencies tested (see figure legend). *Scn8a*^*Clth*^*/Scn8a*^*Clth*^ mice showed an average threshold increase of 10–14 dB SPL. This indicates that *Scn8a*^*Clth*^ mice have mild semi-dominant hearing loss.

To investigate the pathological causes of hearing loss in *Scn8a*^*Clth*^ mice, we examined auditory structures including the outer, middle and inner ears and the peripheral neural auditory pathway. Dissection and X-ray analysis of the middle ear ossicles of 6–10-month-old *Scn8a*^*Clth*^/+ (*n* = 2) and *Scn8a*^*Clth*^/*Scn8a*^*Clth*^ (*n* = 2) mice showed no malformations compared to controls (Fig. 2b). Haemotoxylin and eosin (H&E)-stained sections of middle ears from 8-month-old *Scn8a*^*Clth*^/+ (*n* = 2) and *Scn8a*^*Clth*^/*Scn8a*^*Clth*^ (*n* = 2) mice did not show any differences to controls, with normal middle ear epithelia, intact tympanic membranes and no evidence of otitis media (Fig. 2b). H&E-stained cochlear sections from 6-month-old *Scn8a*^*Clth*^/+ (*n* = 2) and *Scn8a*^*Clth*^/*Scn8a*^*Clth*^ (*n* = 2) mice showed no structural abnormalities in the organ of Corti (Fig. 2b). In addition, scanning electron microscopy (SEM) of cochlear hair cells from 6–7 month-old *Scn8a*^*Clth*^/+ (*n* = 2) and *Scn8a*^*Clth*^/*Scn8a*^*Clth*^ (*n* = 2) mice showed no mispatterning of the stereocilia or degeneration of hair cells, two common pathologies of hearing loss in both mice and humans (Fig. 2b). Gross cochlear structure and outer ear structure in *Scn8a*^*Clth*^/+ and *Scn8a*^*Clth*^/*Scn8a*^*Clth*^ mice were also normal (data not shown).

The lack of an obvious peripheral structural defect (in outer, middle or inner ears) in *Scn8a*^*Clth*^ mice led us to extend our ABR analyses and focus on possible deficits in higher peripheral neural auditory regions. A typical ABR waveform is composed of five peaks, corresponding to electrical signals generated by different components of the peripheral auditory pathway. ABR analysis was performed on sex-matched 5–7-month-old +/+, *Scn8a*^*Clth*^/+ and *Scn8a*^*Clth*^*/Scn8a*^*Clth*^ mice (*n* = 15) using 90 dB SPL tones at 8, 12, 20 and 32 kHz, and peak latencies were statistically analysed for differences (Figs. 2d and 3b,d). Both *Scn8a*^*Clth*^/+ and *Scn8a*^*Clth*^/*Scn8a*^*Clth*^ mice showed no differences in latency of peak 1 (Fig. 3b,d), thought to represent cochlea and cochlear nerve function (Henry 1979). However, *Scn8a*^*Clth*^/*Scn8a*^*Clth*^ mice displayed abnormal waveform morphology from peak 3 compared with control mice: peaks 3 and 4 showed a merged morphology, with peak 4 showing a smaller amplitude than peak 3, and peaks 3–4 and 4–5 interpeak latencies appeared prolonged (Figs. 2d and 3b,d). In addition, peak 5 was often almost absent in *Scn8a*^*Clth*^*/Scn8a*^*Clth*^ mice. Statistical analysis of peak latencies showed that many interpeak measurements were significantly different in *Scn8a*^*Clth*^/+ and *Scn8a*^*Clth*^*/Scn8a*^*Clth*^ mice compared with wild-type mice (Fig. 3b,d). We found that peak 1–5 latency was indeed significantly prolonged, suggesting a recessive model, in response to all frequencies tested (8, 12, 20, 32 kHz). Given the abnormal wave morphology of peaks 3–5, we looked in more detail at peak 3–5 latencies. Peak 3–5 and 4–5 latencies were also significantly prolonged, at different frequencies (Fig. 3b,d), which may reflect different sensitivities of the ABR technique. Of particular interest is the significant prolongation of peak 3–5 latency at 20 kHz, which best fits a dominant inheritance model. As our statistical model did not account for semi-dominant inheritance, examination of the estimate values for this parameter suggests that a semi-dominant model may better fit these results (peak 3–5 latency: *Scn8a*^*Clth*^/+ = +0.525 msec, *Scn8a*^*Clth*^*/Scn8a*^*Clth*^ = +0.842 msec; Fig. 3d). Overall, these results suggest that ABR peak latency between peaks 3 and 5 is prolonged, certainly in *Scn8a*^*Clth*^*/Scn8a*^*Clth*^ mice and probably in *Scn8a*^*Clth*^/+ mice. Peaks 3, 4 and 5 of the ABR are thought to correspond to retrocochlear neural regions of the auditory pathway [probably the superior olivary complex, lateral lemniscus and inferior colliculus (Henry 1979)]. These analyses suggested that peripheral neural auditory function is abnormal in *Scn8a*^*Clth*^ mice. To confirm that the hearing loss in *Cloth-ears* mice was not caused by dysfunction of the cochlear amplifier and, hence (Robles & Ruggero 2001) the outer hair cell function was normal in these mice, we measured DPOAEs in 6-month-old *Scn8a*^*Clth*^*/Scn8a*^*Clth*^ (*n* = 4), *Scn8a*^*Clth*^/+ (*n* = 6) and +/+ (*n* = 3) mice (Fig. 2e). Distortion product otoacoustic emission thresholds were not significantly different between the three genotypes (Fig. 2e), indicating that normal power amplification (Lukashkin *et al.* 2007) of the cochlear mechanical responses is preserved in *Cloth-ears* mice.

### Cloth-ears mice display tremor and paroxysmal movement abnormalities

An additional motor phenotype was identified in *Scn8a*^*Clth*^ mice. Initially, we observed that *Scn8a*^*Clth*^/+ mice displayed an episodic, complex phenotype of extended freezing behaviour, piloerection, a hunched posture and an intermittent side-to-side coarse tremor of the whole body. This phenotype was observed at the earliest in 25-day-old mice. Similarly to the reduced startle response, this phenotype was observed at reduced penetrance (15 of 58 backcross mice), and each component of the phenotype was not always observed in the same mice. However, 25 of 58 backcross mice had either a reduced startle response and/or episodic tremor (43.1%), suggesting that the same underlying mutation was variably affecting both auditory and neurological/neuromuscular function.

In addition, *Scn8a*^*Clth*^*/Scn8a*^*Clth*^ mice showed a more severe phenotype. These mice displayed a continuous and coarse tremor of the head and body, from 8 days old. Tremor was observed in both stationary and walking mice. However, no gait abnormalities were observed in either young or old *Scn8a*^*Clth*^/+ or *Scn8a*^*Clth*^*/Scn8a*^*Clth*^ mice. Interestingly, *Scn8a*^*Clth*^*/Scn8a*^*Clth*^ mice also showed the extended freezing behaviour observed in *Scn8a*^*Clth*^/+ mice, coincident with piloerection and hunched posture. During these freezing episodes, the tremor of *Scn8a*^*Clth*^*/Scn8a*^*Clth*^ mice was also observed to increase in severity and amplitude, strongly resembling the episodic tremor episodes of *Scn8a*^*Clth*^/+ mice. Also, tremor amplitude and the duration of behavioural arrest often increased following behavioural testing, suggesting that stress could be affecting this phenotype. Therefore, we observed two distinct tremor phenotypes in *Scn8a*^*Clth*^ mice, firstly a recessive continuous tremor affecting general posture in *Scn8a*^*Clth*^*/Scn8a*^*Clth*^ mice and secondarily a dominant episodic tremor with behavioural arrest affecting both *Scn8a*^*Clth*^/+ and *Scn8a*^*Clth*^*/Scn8a*^*Clth*^ mice.

**Figure 4:**
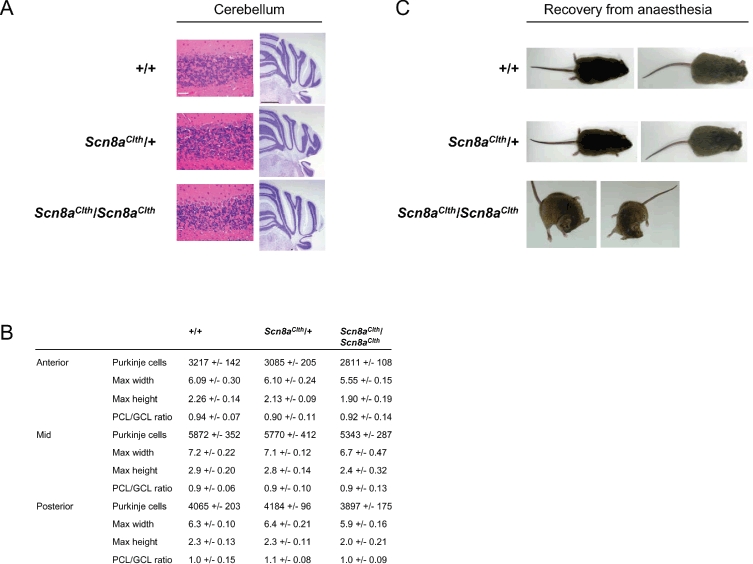
(a) H&E-stained coronal sections of cerebellum from 8-month-old mice show no Purkinje cell loss in any region (scale bar, 50 μm). Histopathology of the cerebellum of 8.5-month-old mice using cresyl violet on coronal sections shows no neuronal mislocalization (scale bar, 1 mm). (b) Area and layer measurements and Purkinje cell counts of *Scn8a*^*Clth*^ mice. Although *Scn8a*^*Clth*^*/Scn8a*^*Clth*^ cerebellums are slightly smaller and show slightly reduced Purkinje cell counts compared with +/+ and *Scn8a*^*Clth*^/+, the Purkinje cell layer (PCL) to granule cell layer (GCL) ratio is normal, indicating normal overall cerebellar structure. Area measurements are in millimetres. (c) Abnormal dystonic postures displayed by *Scn8a*^*Clth*^*/Scn8a*^*Clth*^ mice during recovery from anaesthesia were not seen in +/+ or *Scn8a*^*Clth*^/+ mice. Postures were maintained for up to 1 min.

We assessed if the tremor and motor abnormalities in *Scn8a*^*Clth*^ mice could be associated with muscular, cerebellar or other tissue changes. Sections of semimembranosus and quadriceps muscles from 8.5-month-old *Scn8a*^*Clth*^/+ and *Scn8a*^*Clth*^*/Scn8a*^*Clth*^ mice (*n* = 4) showed no differences to wild-type muscles in H&E-stained sections. Specifically, there was no evidence of degenerative or regenerative changes (fibre size asymmetry, pyknotic nuclei, centrally nucleated fibres; data not shown). We also examined H&E-stained cerebellar sections from 8.5-month-old *Scn8a*^*Clth*^/+ and *Scn8a*^*Clth*^*/Scn8a*^*Clth*^ mice (*n* = 4) that showed no indication of Purkinje cell loss or disorganization of the trilaminar structure (Fig. 4a). To quantify any potential abnormalities of the mutant cerebellum further, we examined sections of 8.5-month-old *Scn8a*^*Clth*^/+ and *Scn8a*^*Clth*^*/Scn8a*^*Clth*^ mice using cresyl violet staining (*n* = 4) (Fig. 4a). Cerebellar dimension and layer measurements and Purkinje cell counts showed that although cerebellum from *Scn8a*^*Clth*^*/Scn8a*^*Clth*^ mice are slightly smaller than +/+ and *Scn8a*^*Clth*^/+ animals and show a slightly reduced number of Purkinje cells, the ratio of the PCL to GCL width is normal, suggesting the overall structural development and maintenance of the cerebellum are not affected in *Scn8a*^*Clth*^*/Scn8a*^*Clth*^ mice (Fig. 4b). Furthermore, no neuronal mislocalization was observed and the lamination of the cortex and hippocampus appeared normal in all sections examined, thus the small *Scn8a*^*Clth*^*/Scn8a*^*Clth*^ cerebellar differences are likely to reflect the overall small size of the mice (Fig. 5a,c). H&E-stained sections of 33 other tissues and organs from 8.5-month-old *Scn8a*^*Clth*^/+ and *Scn8a*^*Clth*^*/Scn8a*^*Clth*^ mice (*n* = 4) showed no histological differences to wild-type mice (data not shown). Peripheral (sciatic) nerves showed no abnormal myelination and spinal cord sections showed no abnormalities (data not shown).

Lastly, we noticed that the response to anaesthesia in *Scn8a*^*Clth*^*/Scn8a*^*Clth*^ mice was abnormal. During recovery from anaesthesia (ketamine/medetomidine) after ABR testing, *Scn8a*^*Clth*^*/Scn8a*^*Clth*^ mice displayed abnormal dystonic postures (*n* = 5) that were not observed in *Scn8a*^*Clth*^/+ or +/+ mice (Fig. 4c), and a longer time to regain normal movement (data not shown).

### Behavioural abnormalities in Cloth-ears mice

The unusual motor symptoms and freezing behaviour in *Scn8a*^*Clth*^ mice led us to investigate the gross phenotype of *Scn8a*^*Clth*^ mice by detailed phenotypic and behavioural testing of +/+, *Scn8a*^*Clth*^/+ and *Scn8a*^*Clth*^*/Scn8a*^*Clth*^ littermate mice at 6, 10 and 18 weeks of age (*n* = 73). These time-points spanned the ages at which these phenotypes appeared to develop in *Scn8a*^*Clth*^ mice. Data was statistically analysed for a genotype effect. To adjust for confounding variables, continuous numeric data were analysed under a linear model using anova and multivariate *F*-test; binary data were analysed under a generalized linear model and ordinal data were analysed under a proportional odds logistic regression model (see *Methods* for further information). Parameters showing a significant difference were further analysed to determine the best fit of inheritance (dominant or recessive) (see *Methods*). This analysis showed that *Scn8a*^*Clth*^ mice show significant dominant, recessive, and probably semi-dominant abnormalities in growth, neurological and motor function and behaviour. The weight of *Scn8a*^*Clth*^ mice was significantly less than wild type at all ages tested (Fig. 5a,c), with statistical analysis suggesting both recessive (6 weeks, 10 weeks) and dominant (18 weeks) inheritance. Estimate values for *Scn8a*^*Clth*^/+ mice were intermediate between wild-type and *Scn8a*^*Clth*^*/Scn8a*^*Clth*^ values (Fig. 5c), suggesting that growth retardation is likely to be semi-dominant. Grip strength in (1) forelegs and (2) all legs was not different in *Scn8a*^*Clth*^/+ mice at any age tested (Fig. 5a). *Scn8a*^*Clth*^*/Scn8a*^*Clth*^ mice were not tested using the grip strength test for welfare reasons. Ability to perform the wire manoeuvre test was recessively impaired in *Scn8a*^*Clth*^ mice at 6, 10 and 18 weeks of age (Fig. 5a,c). *Scn8a*^*Clth*^ mice also showed recessive increased tendency to limb grasp (Fig. 5a,c). Trunk curling during tail suspension was not successfully analysed statistically because of experimental imbalance (Fig. 5a), but individual test results showed that most *Scn8a*^*Clth*^*/Scn8a*^*Clth*^ mice displayed abnormal trunk curling behaviour, instead of the splayed limbs of wild-type mice (data not shown). The toe-pinch reflex was recessively impaired in *Scn8a*^*Clth*^ mice at all ages tested (Fig. 5a,c). However, proximal reflex tests of corneal, pinna and reaching reflexes were all normal (Fig. 5a).

We assessed general behaviour by observing mice undisturbed for 1 min (see *Methods*). *Scn8a*^*Clth*^ mice showed a recessive increased tendency to freeze during testing at all ages tested (Fig. 5a,c) and a recessive increased duration of freezing after the clickbox test (where wild-type mice normally freeze for 1–2 seconds) at 10 and 18 weeks (Fig. 5a,c). No significant differences between *Scn8a*^*Clth*^ mice and wild-type mice were found in facial twitching, muscle spasms of body, piloerection, shaky or unsteady movement, abnormal postures, repetitive nosepoking, visible hyperactivity or excessive rearing (data not shown).

The freezing behaviour in both *Scn8a*^*Clth*^/+ and *Scn8a*^*Clth*^/*Scn8a*^*Clth*^ mice raised the possibility that *Scn8a*^*Clth*^ mice could suffer high anxiety. We tested anxiety and locomotion in 5–6-month-old sex-matched *Scn8a*^*Clth*^/+ and *Scn8a*^*Clth*^*/Scn8a*^*Clth*^ mice (*n* = 39) using the open-field and light-dark box paradigms. *Scn8a*^*Clth*^ mice did not show any significant differences in any parameters tested in the open-field test, indicating no observed anxiety or locomotion defects compared with wild-type mice (Fig. 5b). However, the latency of first entrance into the lit half of the light-dark box was significantly recessively reduced in *Scn8a*^*Clth*^*/Scn8a*^*Clth*^ mice (Fig. 5b,d).

## Discussion

We report that a novel ENU-induced mouse mutant, *Cloth-ears* (*Scn8a*^*Clth*^), shows hearing loss with peripheral neural auditory impairment as well as paroxysmal motor symptoms and tremor, and that a missense mutation in *Scn8a* underlies this phenotype. The data suggests that SCN8A is critical for normal peripheral auditory function and may play a role in the pathogenesis of paroxysmal motor disorders.

### Scn8a^*Clth*^ mice are a model of peripheral neural hearing loss

We did not detect any morphological or functional pathology in middle or inner ears from *Scn8a*^*Clth*^ mice by histology, ABR and DPOAE analysis, showing that the *Scn8a*^*Clth*^ mutation does not affect middle ear or cochlear function. However, ABR analysis showed that *Scn8a*^*Clth*^ mice have abnormal morphology of peaks 3–5 and lengthened peak latencies. Peaks 1–5 of the ABR in mice are generally considered to arise approximately from the cochlear origins (peak 1), from the cochlear nucleus (peak 2), the superior olivary complex (peak 3), the lateral lemniscus (peak 4) and the inferior colliculus (peak 5) (Henry 1979). Our findings suggested that the auditory deficit in *Scn8a*^*Clth*^ mice is caused by a peripheral neural defect originating from the superior olivary complex, lateral lemniscus or inferior colliculus (peaks 3, 4 and 5, respectively), although these generators of the ABR are still contested (Henry 1979). This suggests that SCN8A is involved in auditory nerve and peripheral neural auditory function. SCN8A is localized to the organ of Corti and cochlear ganglionic axons in the mouse, and has been shown to be involved in the generation and regeneration of action potentials in cochlear ganglion cells (Hossain *et al.* 2005). Interestingly, a role for *Scn8a* in the central auditory pathway was previously suggested (Chen *et al.* 1999) by observation of greatly reduced spontaneous bursting activity of neurons in the dorsal cochlear nucleus (DCN) in brain slices from *Scn8a*^*med*−*J*^/*Scn8a*^*med*−*J*^ and *Scn8a*^*med*−*jo*^/*Scn8a*^*med*−*jo*^ mice. However, no hearing impairment has been previously reported for any *Scn8a* mouse mutants, and indeed hearing in dystonic *Scn8a*^*med*−*J*^/*Scn8a*^*med*−*J*^ (*Scnm1*^*R*^*/Scnm1*^*R*^) mice was normal (Koay *et al.* 2002). Therefore, it is possible that the *Cloth-ears* mutation represents a unique gain-of-function allele in the auditory system. Thus, reassessment of other *Scn8a* alleles for subtle hearing deficits would be merited. Further histological and functional analysis of the auditory pathway in *Scn8a*^*Clth*^ mice would identify which structures are primarily, and secondarily, affected. The *Cloth-ears* mutant is therefore the first evidence that mutation in *Scn8a* can result in hearing impairment in mice.

### Motor deficits in Scn8a^*Clth*^/Scn8a^*Clth*^ mice

*Scn8a*^*Clth*^*/Scn8a*^*Clth*^ mice begin to display a continuous tremor at 8 days old, and later, show impairment at the wire manoeuvre test and the distal toe-pinch reflex, that suggests muscle weakness and distal sensory loss is occurring in these mice. The onset of skeletal muscle weakness or paralysis in the lethal *Scn8a* mutants is also from around 8 days old (Buchner *et al.* 2004; Duchen & Strich 1966; Lane 1976). Moreover, Garcia *et al.* (1998) showed that SCN8A was responsible for the threefold increase in sodium current between postnatal day 0 and day 8 in mouse motor neurons, and suggested that this was caused by postnatal upregulation of *Scn8a.* Thus, the onset of motor symptoms in *Scn8a*^*Clth*^*/Scn8a*^*Clth*^ mice correlates with the reported upregulation of *Scn8a* in motor neurons. In contrast to almost all published *Scn8a* mouse mutants, we did not observe degenerative changes in skeletal muscle, cerebellum or any gait defects or paralysis in *Scn8a*^*Clth*^*/Scn8a*^*Clth*^ mice. Particularly, this is markedly different to a non-lethal *Scn8a* mutant that shows a tremor: *Scn8a*^*med*−*jo*^/*Scn8a*^*med*−*jo*^ mice have a marked loss of cerebellar Purkinje cells (Dick *et al.* 1985). A functional but mild impairment of one or more of these tissues could therefore underlie the motor deficits in *Scn8a*^*Clth*^*/Scn8a*^*Clth*^ mice. Functional abnormalities previously identified in *Scn8a* mouse mutants include a loss of resurgent sodium current and disrupted firing patterns in cerebellar Purkinje cells (Raman *et al.* 1997), reduced conduction velocity and a prolonged refractory period in motor neurons (Duchen & Stefani 1971) and a failure of muscle fibres to show action potentials in response to nerve stimulation (Duchen & Stefani 1971). Electromyography/electophysiological analysis of muscle, motor neurons and cerebellar output would be needed to delineate the origins of tremor and muscle weakness in *Scn8a*^*Clth*^*/Scn8a*^*Clth*^ mice.

### Behavioural phenotypes in Scn8a^*Clth*^/Scn8a^*Clth*^ mice

Both *Scn8a*^*Clth*^/+ mice and *Scn8a*^*Clth*^*/Scn8a*^*Clth*^ mice display an episodic tremor with behavioural arrest phenotype. This similarity of phenotype and its infrequent nature suggests that this is a dominant episodic phenotype occurring under particular external or internal conditions that could be seizure- or dystonia-like. Genetic and non-genetic factors are known to moderate some paroxysmal (episodic) neurological and motor disorders in mice and humans. For example, myotonia in the human disorder paramyotonia congenita caused by mutation in the *SCN4A* gene (OMIM: #168300) is aggravated and/or induced by exposure to cold temperatures (OMIM). Indeed, even *Scn8a* has recently been reported to modulate the severity of seizures in a mouse mutant of *Scn2a* (Martin *et al.* 2007). We tested a small cohort of *Scn8a*^*Clth*^/+ mice to see if handling or auditory testing induced tremor episodes, but only a small increase in tremor occurrence was observed (data not shown), indicating that these stimuli were not major inducers of tremor. Robust testing of the effect on tremor occurrence of other environmental factors such as temperature, and crossing to different genetic backgrounds, would be useful in isolating the precipitators of tremor episodes in these mice.

Alternatively, it could be that *Scn8a*^*Clth*^/+ and *Scn8a*^*Clth*^/*Scn8a*^*Clth*^ mice have heightened anxiety, leading to increased freezing (behavioural arrest). We found that the incidence and duration of freezing was significantly increased in *Scn8a*^*Clth*^*/Scn8a*^*Clth*^ mice by visual observation. However, the decreased time of latency of first move into the light-half in the light-dark paradigm shown by *Scn8a*^*Clth*^*/Scn8a*^*Clth*^ mice could suggest that these mice show lessened anxiety. Open-field analysis did not show any differences in locomotion and anxiety measures of *Scn8a*^*Clth*^ mice. Furthermore, *Scn8a*^*Clth*^*/Scn8a*^*Clth*^ mice displayed a greater frequency of trunk curling and limb grasping, which are often displayed by mouse mutants with neurological defects including *Scn8a*^*med*−*TgA*4*Bs*^/*Scn8a*^*med*−*TgA*4*Bs*^ mice (Kohrman *et al.* 1995). SCN8A is highly expressed in the hippocampus (Caldwell *et al.* 2000), which is involved in fear-related behavior. Recently, it has been reported that mice heterozygous for a null allele of *Scn8a* show evidence of ’emotional’ behavioural defects, including increased freezing and avoidance of the centre in open-field tests (McKinney *et al.* 2008). This study indicates that, for some behavioural phenotypes, the null heterozygote is more severe than the *Cloth-ears* allele described here. In addition, the only family identified with a *SCN8A* mutation show neuropsychological impairment (Trudeau *et al.* 2006). A positive association between *SCN8A* variants and suicide attempts in humans has also been reported (Wasserman *et al.* 2005). It would be interesting to assess if a seizure-like or anxiety phenotype underlies the behavioural abnormalities in *Scn8a*^*Clth*^ mice by electroencephalography and further behavioural testing.

### Mechanism of SCN8A dysfunction in Scn8a^*Clth*^ mice

Complementation testing showed that the Asp981Val mutation in SCN8A underlies the *Scn8a*^*Clth*^ phenotype. Voltage-gated sodium channels are transmembrane proteins that allow fast influx of sodium ions into excitable cells in response to membrane depolarization, and are responsible, among other functions, for generating and conducting action potentials (reviewed in Ogata & Ohishi 2002). The widespread expression of *Scn8a* in the central and peripheral nervous system (Caldwell *et al.* 2000) and the critical function of SCN8A in generating sodium currents in neurons (Cummins *et al.* 2005; Garcia *et al.* 1998; Raman *et al.* 1997) suggest that functional defects in neuronal excitability could be responsible for the auditory defects, tremor and neurological/behavioural defects of *Scn8a*^*Clth*^ mice. It is possible that the Asp981Val amino acid substitution causes a change in SCN8A channel kinetics or sodium current properties. Electrophysiology on another *Scn8a* mouse mutant with tremor [*Scn8a*^*med*−*jo*^*/Scn8a*^*med*−*jo*^ (Ala1071Thr)] showed that the *Scn8a*^*med*−*jo*^ mutation caused a shift in the voltage dependence of SCN8A channel activation (Kohrman *et al.* 1996), which could decrease neuronal excitability and thus result in ataxia and tremor. Additionally, the close proximity of the Asp981Val amino acid change to the predicted end of the D2-S6 segment (Fig. 1d) suggests that it is possible that this residue could be involved in the function of domain 2. Indeed, several mutations in the D2-S6 segment of the rat *Scn2a* have been shown to affect channel kinetics, including altering the voltage dependence of activation and inactivation, and causing incomplete inactivation (Yarov-Yarovoy *et al.* 2002). Also, we found that *Scn8a*^*Clth*^*/Scn8a*^*Clth*^ mice showed abnormal dystonic postures after anaesthesia, that were not seen in *Scn8a*^*Clth*^/+ and wild-type mice. Interestingly, residues in the D2-S6 segment have been shown to be involved in the binding and action of anaesthetics to VGSCs (Kondratiev & Tomaselli 2003; Wang *et al.* 2001). Additionally, several amino acid mutations in other VGSC α-subunit genes that lie in very close proximity to the Asp981 amino acid have been identified in four human disorders. Functional consequences of these mutations include changes in fast inactivation and persistent currents (Green *et al.* 1998; Schwartz *et al.* 2000). Intriguingly, symptoms in these four human disorders are episodic, and believed or known to be precipitated by external or internal stimuli: the paroxysmal muscular disorders, paramyotonia congenita/myotonia congenita and myotonia fluctuans (*SCN4A*) (McClatchey *et al.* 1992; Ricker *et al.* 1994); near-sudden infant death syndrome (SIDS) caused by the cardiac condition long QT syndrome type 3 (LQT3) (*SCN5A*) (Schwartz *et al.* 2000) and severe myoclonic epilepsy of infancy (SMEI) (*SCN1A*) (Claes *et al.* 2001; Escayg *et al.* 2000). Thus, we predict that the *Scn8a*^*Clth*^ Asp981Val mutation could alter SCN8A channel kinetics or sodium current properties in *Scn8a*^*Clth*^ neurons, which could be assessed *in vitro* and *in vivo*.

### The Scn8a^*Clth*^ mouse is a resource for studying human disease

The *Scn8a*^*Clth*^ mouse is a novel mutant that links peripheral neural hearing loss with tremor and paroxysmal motor symptoms. A small number of mouse mutants with continuous tremor and hearing impairment have been reported (MGI, OMIM), including several other ion channel mutants [*Atp2b2* (plasma membrane calcium-transporting ATPase 2): deafwaddler^2J^ (Noben-Trauth *et al.* 1997) and deafwaddler^3J^ (McCullough & Tempel 2004); *Spnb4* (spectrin beta 4): quivering (Parkinson *et al.* 2001); *Kcnj10* (ATP-sensitive inward rectifier potassium channel 10) (Marcus *et al.* 2002; Neusch *et al.* 2001)]. However, no mutant mice with hearing loss and paroxysmal tremor have been reported. Around 12 human disorders feature both hearing impairment and tremor (OMIM). Essential tremor patients show significantly increased occurrence of high-frequency sensorineural hearing loss (Ondo *et al.* 2003); however, a recent screen of patients with essential tremor did not detect any variants of *SCN8A* (Sharkey *et al.* 2008). Sensorineural deafness and tremor are also seen in the autosomal dominant demyelinating Charcot-Marie-Tooth disease type 1E (OMIM) and in 18q deletion dystonia syndrome (Gordon *et al.* 1995). The paroxysmal symptoms in *Scn8a*^*Clth*^ mice and in human disorders caused by mutations in other *SCN*α-subunit genes suggest that mutation in *SCN8A* could also contribute to paroxysmal disorders in humans. It will be intriguing to consider *SCN8A* as a candidate gene for peripheral neural hearing loss and paroxysmal neurological or neuromuscular disorders in humans.
